# A Randomized Controlled Pilot Study to Assess Effects of a Daily Pistachio (Pistacia Vera) Afternoon Snack on Next-Meal Energy Intake, Satiety, and Anthropometry in French Women

**DOI:** 10.3390/nu11040767

**Published:** 2019-04-02

**Authors:** Arianna Carughi, France Bellisle, Anestis Dougkas, Agnès Giboreau, Mary Jo Feeney, Jennette Higgs

**Affiliations:** 1 American Pistachio Growers, Fresno, CA 93720, USA; 2 Nutri Psy Consult, 75013 Paris, France; bellisle.france@gmail.com; 3 Institut Paul Bocuse Research Centre, BP 25-69130 Ecully CEDEX, France; anestis.dougkas@institutpaulbocuse.com (A.D.); agnes.giboreau@institutpaulbocuse.com (A.G.); 4 Consultant, California Agricultural Boards, Los Altos Hills, CA 94024, USA; mj@feeney.us.com; 5 Food To Fit Ltd., London KT1 4AE, UK; jennette@foodtofit.com

**Keywords:** pistachio, satiety, appetite, snack, energy intake, visual analogue scales (VAS)

## Abstract

Including nuts in the diet improves appetite control and does not lead to weight gain. However, for pistachios, evidence from randomized intervention studies is limited and there are no data on the effect of pistachios on satiety. The objective of this study was to assess the effect of daily consumption of pistachios as an afternoon snack on satiety, self-reported energy, self-reported nutrient intake, body weight, and body composition. This randomized controlled pilot study included two parallel groups of 30 healthy French women, in a free-living setting. For four weeks, groups were instructed to consume either 56 g (1318 kJ) of pistachios or 56 g of isoenergetic/equiprotein savory biscuits as an afternoon snack. Evening energy intake, changes in anthropometric measures, and daily intake of energy and selected nutrients were assessed. Visual analogue scales (VAS) were used to rate hunger, fullness, desire to eat, and prospective consumption. Satiety effects were not different between groups, as assessed by evening energy intake or VAS scores. Consuming pistachios or biscuits had no impact on body weight. Thiamin, vitamin B6, copper, and potassium intakes were significantly higher in the pistachio group. Consuming pistachios or biscuits as an afternoon snack resulted in similar post-snack food intake and subjective feelings of satiety. A daily pistachio snack for a month did not affect body weight or composition but it did improve micronutrient intake.

## 1. Introduction

The high prevalence of overweight and obesity worldwide is among the more significant public health concerns of the twenty-first century. Therefore, the role of healthy nutritious food choices, controlled energy intake, and physical activity are at the core of nutrition policy messages emphasized by health agencies around the world. Given that snacking, which is defined as eating apart from main meals [1], can influence energy and nutrient intake, the choice of snack food may affect weight gain and diet quality [2]. In most countries, dietary guidelines mention nuts, including pistachios, as healthy foods, consumption of which is recommended [3,4]. Nuts, as important components of the Mediterranean diet, have been shown to provide a multitude of health benefits including reducing the risk of cardiovascular disease and type 2 diabetes mellitus (T2D) and lowering the risk of all-cause mortality [5,6,7,8]. These benefits are thought to be due to the healthful nutrient profile of nuts and their abundance of health protective bioactive compounds [9]. Therefore, nuts such as pistachios may be considered a healthful snack option.

Nuts, including pistachios, are nutrient- and energy-dense foods. Although low in saturated fatty acids and high in mono- and polyunsaturated fatty acids, there is a perception that including nuts in the diet on a regular basis will result in weight gain [10]. However, the evidence does not support this perspective. Regular nut intake has been inversely associated with adiposity and weight gain in many large epidemiological studies. For example, in Harvard’s prospective Nurses’ Health Study, women who consumed nuts more than two times per week gained less weight and had a lower risk of obesity than those who seldom ate nuts [11]. In an analysis that combined three separate cohorts of adult men and women who were followed for nearly twenty years, intake of nuts was associated with weight loss (−0.26 kg) over a four-year period [12]. In the United States, researchers analyzed the National Health and Nutrition Examination Survey (NHANES) cross-sectional database and found that tree nut consumption was associated with lower BMI and waist circumference [13].

Intervention studies, robust sources of evidence for establishing causal relationships, are consistent with these findings. The PREDIMED study (PREvención con Dieta MEDiterránea) assessed the effect of a low-fat diet, a Mediterranean diet supplemented with olive oil and a Mediterranean diet supplemented with nuts, on cardiometabolic risk outcomes [7]. While changes in body weight did not differ across diet groups, participants assigned to supplemental nut consumption showed a significant decrease in central adiposity. A recent meta-analysis of 33 randomized clinical trials found no association between consumption of nuts and body weight, BMI, or waist circumference [14]. Looking at pistachios specifically, four randomized, controlled clinical studies have evaluated the effect of including pistachios in the diet on body weight [15,16,17,18]. A twelve-week weight-loss study in overweight or obese individuals who consumed either 53 g/d pistachios or 56 g/d of salted pretzels as an afternoon snack showed a significant reduction in BMI in the pistachio-supplemented group [15]. This reduction was greater than that observed in the pretzel-supplemented group (24% vs. 22% of BMI). A recent free-living cross-over study in college aged healthy women found no change in body weight after a ten-week pistachio supplementation at 20% of energy [17]. Furthermore, several trials have evaluated the effect of pistachio consumption on body weight and/or BMI as a secondary outcome [19,20,21,22,23]. In all of the trials, participants consumed pistachios as at least 15% of their daily energy intake. There was no significant effect on body weight and/or BMI seen compared to participants on the control diets [19,20,21,22,23].

Several biological mechanisms have been suggested to explain why, despite a relatively high caloric and fat content, consumption of nuts is not associated with weight gain [24,25]. Nuts are rich in unsaturated fats, which may have a greater thermogenic effect than saturated fats, resulting in less fat storage [26]. Unsaturated fats may also influence satiety [27]. In addition, not all of the fat in nuts is absorbed following consumption, resulting in an overestimation of their caloric contribution to the diet. This has been demonstrated in walnuts, almonds, and pistachios [28,29,30]. Therefore, nuts may provide less metabolizable energy in vivo than that calculated by proximate analysis and standardized Atwater factors. Nuts, which are energy dense and high in fiber and protein, may promote satiety (sensation of fullness that inhibits further eating in the postprandial period [31]) resulting in fewer calories consumed at subsequent eating occasions.

Satiety can be assessed using various methods. Subjective sensations of appetite can be followed, over time, after the consumption of a food or a meal using tools such as the visual analogue scales (VAS) that allow a person to score the intensity of hunger, fullness, and desire to eat experienced at specific times for several hours following one eating event [32]. Satiety effects can also be measured by assessing energy intake at the next eating occasion.

Satiety effects of pistachios either in terms of next meal energy intake or satiety sensations remain unexplored. A recent meta-analysis of randomized clinical studies on the effects of nuts on energy intake, hunger, and fullness included 31 trials [33]. Pooled estimates of clinical trials showed increased daily energy intake in obese/overweight but not in normal weight participants, and decreased hunger following nut consumption. The nuts tested included almonds, walnuts, peanuts, hazelnuts, and pecans. None of the trials were conducted on pistachios. However, pistachios are unique among common nuts as they are usually purchased in-shell and so require shelling. It has been shown that participants consumed fewer in-shell pistachios compared to shelled pistachios, during a set time interval, either because of the additional time needed to shell the pistachios or the extra volume perceived when viewing the pistachio shells left in sight after consumption [34,35]. Therefore, the effect of pistachios on satiety measures cannot be extrapolated from those of other nuts.

A crucial aspect of satiety effects is their stability over time. In order for acute satiety effects to exert any impact on body weight, such effects must be maintained over repeated exposures. Indeed, regulatory mechanisms for energy balance that adjust energy intake to energy expenditure normally act to compensate for fluctuations in food energy content [36]. Studies on almonds [37,38], peanuts [39], and walnuts [40] suggested that nuts have a high satiety value. Therefore, it is important to establish if any satiety effect that might be observed following the acute consumption, under specific intake circumstances, is maintained over repeated experiences of such intake. Only one intervention study has assessed this in walnuts [41] and two in almonds [42,43]. There has not been any studies that have assessed satiety effects over time in pistachio intervention studies.

The primary objective of this four-week study in healthy French women was to investigate the effect of consuming pistachios versus an isoenergetic/equiprotein control food as a “goûter” on satiety, as assessed by next-meal energy intake, and subjective ratings of hunger, fullness, desire to eat, and prospective consumption. The goûter is a traditional small meal ingested in the middle of the afternoon by a large proportion of the French population [4]. It can be considered a snack because of its small size, relatively irregular nature, ready-to-eat options, and ingestion outside of main meals. Secondary aims were to investigate the impact of the intervention on anthropometry and body composition, and selected micronutrient intake. Therefore, the hypotheses of this study are that a daily intake of pistachios as an afternoon goûter under free-living conditions suppresses energy intake during the evening and enhances satiety sensations in comparison to a control food, and furthermore, does not cause weight gain and increases micronutrient intake.

## 2. Materials and Methods

### 2.1. Subjects

Volunteers were recruited locally (Nantes area) using the clinical site database of volunteers, newspaper advertisements, and campus announcements. Each volunteer received 200E for participating in the study. They were healthy, sedentary women, 18–50 years of age (N = 60) who met the following inclusion criteria: BMI ≥ 18. 5 kg/m^2^ ≤ 25 kg/m^2^; weight stable within ±2 kg in the last 3 months; a “restrained eating” score of < 17 or a ”disinhibition” score < 13, as revealed by the Three Factor Eating Questionnaire (TFEQ) [44]; not taking supplements or herbal/botanicals or pharmaceutical products claimed to suppress appetite; on stable contraceptive medication; not undergoing menopause; not perimenopausal or undergoing disrupted menstrual cycle patterns; eating a regular goûter at least three times per week; and with no known allergies to nuts or control food. Regular consumers of pistachios, other tree nuts, or peanuts (defined as consuming nuts more than three times a week) were excluded from the study. Only women were recruited for the study to limit heterogeneity. Women are more weight-concerned than men [45] and could be more reluctant to follow the recommendations to include nuts in the daily diet.

The CPP Ouest IV of Nantes (ethics review board) approved the study for implementation. This study was carried out in accordance with the Good Clinical Practice standards, the declaration of Helsinki and current French regulation (Code de Santé Publique, Titre II du livre Premier). At screening, women received all information concerning the study, including the objective, design, duration, any risks, and the benefits of the study. Written informed consent was obtained from all subjects before their participation in the study.

### 2.2. Study Design and Protocol

This was a pilot randomized, controlled, open trial. Subjects were randomized into two parallel groups, experimental and control, according to a pre-determined randomization list in chronological sequence of inclusion. The block randomization table was generated using SAS^®^ software (version 9.3, SAS Institute Inc., Cary, NJ, USA). For four weeks the experimental group consumed 56 g (1318 kJ) of roasted, lightly salted in-shell pistachios as an afternoon snack, while the control group consumed an isoenergetic/equiprotein commercially available savory cheese biscuit. Because of the different foods consumed by the subjects, no blinding procedure was possible. The sample size (60) is consistent with published recommendations for evaluating the effect of foods on satiety [46]. This study was designed as a pilot study as there were no available data about expected size effect on energy intake, after an afternoon goûter, or the associated variability. Furthermore, the impact of the act of shelling pistachios on measures of satiety in this setting were not known.

**Figure 1:**
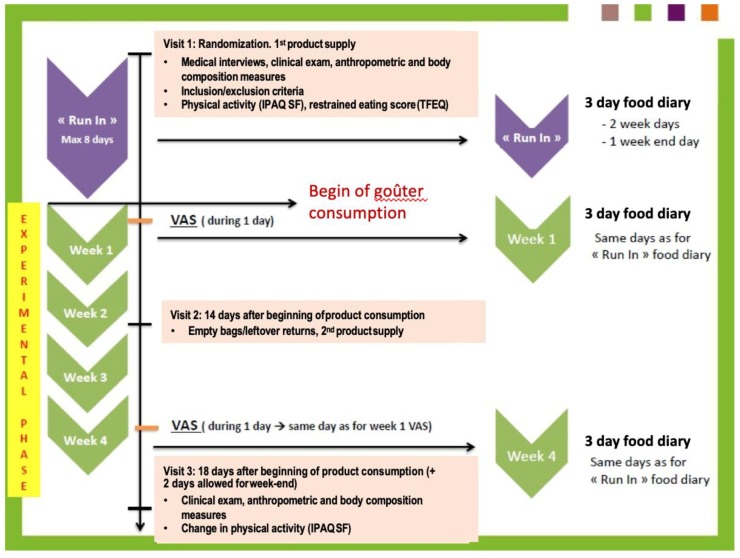
Experimental design.

Each subject came to the center for three visits: visit 1 (run-in), inclusion and randomization visit when they were supplied with test foods; visit 2, follow-up visit when they received a second supply of test foods; and visit 3, end of study visit. The final anthropometry and body composition measurements were taken at this visit (week 4). Anthropometry and body composition measurements, medical history, assessment of eating habits, and explanation of study protocol took place during visit 1. The run-in period was a maximum of eight days between visit 1 and the start of test food consumption (week 1). During this time, they did not consume the study goûters and followed their usual food and lifestyle habits. During the experimental phase (four weeks), subjects replaced their usual daily goûter with a fixed pre-packaged serving of either pistachios or biscuits. (see Figure 1).

On the first day of the first week, and one day of the fourth week, subjects were asked to report their sensations of hunger and satiety at predetermined times using the visual analogue scales (VAS). Subjects were provided with food diaries to complete on three days (one of which was a weekend day) on three occasions during the study (at run-in, week 1, and week 4). The food diary days were randomly assigned by the study organizers in advance, and subjects were asked to complete their diaries on the same days throughout the study. Subjects were given clear directions for recording volumes/measures and for carefully recording the time when meals and snacks were consumed. Completed diaries were collected at each subsequent visit.

Each subject was reached by phone twice during the experimental phase in order to stimulate compliance and record any adverse event. In addition, text messages or e-mails were sent twice weekly to remind subjects to consume their goûters. Throughout the study, participants kept a consumption diary, recording the beginning and end times of the goûter consumption. This was used to calculate the time it took for participants to consume the goûter. Subjects were instructed to bring back unfinished, empty, or unused bags at the second and third visit. Compliance was checked for every subject at these dates by verifying their consumption diary and weighing each bag brought back. The compliance of the period between the first and second visit, between the second and third visit, and the global compliance mean of each period compliance) were calculated and expressed as percentages. A day compliance lower than 75% was considered a deviation to the protocol.

### 2.3. Test and Control Foods (Goûters)

The experimental group received roasted, lightly salted in-shell pistachios (American Pistachio Growers, Fresno, CA, USA), supplied in 56 g bags (1318 kJ). This amount was chosen in order to expose participants to a fixed 15% of the daily mean energy intake in French adult women (total intake, including alcohol, is 7760 ± 1780 kJ) [4]. The control group received 56 g bags of “Gouda aperitif biscuits”, of comparable protein and energy content (1318 kJ). Table 1 lists nutrient content, showing that pistachios have notably higher levels of potassium, copper, and vitamin B6 than the savory biscuits.

### 2.4. Measurements

#### 2.4.1. Energy and Nutrient Intake and Physical Activity

Energy and nutrient intake were evaluated with a three-day food diary. Data collected in this food diary were analyzed by a certified dietitian using standard procedures on validated software (Nutrilog SAS, Marans, France). Total energy intake (total energy during the day including energy from goûter), energy intake during the evening (energy intake at dinner + post-dinner), and total daily intake of thiamin, vitamin B6, copper, manganese, phosphorous, and potassium were calculated from the food diaries. The physical activity score (in MET-min/week) was assessed using the International Physical Activity Questionnaire (IPAQ) short form [47].

#### 2.4.2. Anthropometric Measures

Body weight (kg) was calculated to the nearest 0.1 kg with the subject being in a fasted state and wearing light clothes and no shoes. Height was measured to the nearest 0.1 cm with a wall-mounted stadiometer (Seca Limited). The waist and hip circumference (cm) were measured by qualified trained professionals using standardized validated procedures. The waist/hip circumference was calculated using the formula, ratio = waist circumference (cm)/hip circumference (cm). The fat/lean body mass ratio was calculated using the formula: ratio = fat body mass (%)/lean body mass (%). The body composition (% fat body mass and % lean body mass) was determined by performing a bioelectrical impedance analysis using a Bodystat 1500 device (Ballakaap, British Isles) according to standardized validated operating procedures [48]. These measures were taken at run-in and at week 4. Effort was spent to ensure that the same operator took the measurements at run-in and week 4 for the same subject.

#### 2.4.3. Subjective Measures Using Visual Analog Scales (VAS)

Subjects were asked to rate their sensations of hunger, thirst, fullness, desire to eat, and prospective consumption by answering questions on the VAS. The VAS consisted of 100 mm long horizontal lines anchored at both ends with phrases expressing extreme levels of one sensation (for example, “not hungry at all” and “extremely hungry”). This approach has been shown to be valid, reliable, and reproducible [49]. Ratings were obtained immediately before breakfast (T1), immediately after breakfast (T2), immediately before lunch (T3), immediately after lunch (T4), immediately before and immediately after goûter (T5 and T6, respectively), at 30 (T7), 60 (T8), 90 (T9), and 120 (T10) minutes after goûter, immediately before (T11), and immediately after (T12) dinner.

### 2.5. Statistical Analyses

A mixed model analysis of covariance (ANCOVA) for repeated measures (SAS^®^ PROC MIXED, SAS Institute Inc., Cary, NC, USA) was used to estimate the effects of the pistachio goûter on total energy intake, energy intake during the evening, nutrient intake, and the time subjects took to consume the goûter. Treatment (pistachio vs. control), weeks (week 1 and week 4). and treatment x week interaction were included as fixed effects. Subject was treated as random effect while week was included as repeated effect. The normality of residuals and data distribution were tested in all models using standard diagnostics. Changes in body composition and anthropometric measures between run-in and week 4 (endpoint) were analyzed as above. The VAS individual scores of hunger, fullness, desire to eat, thirst, and prospective consumption were analyzed using an ANOVA for repeated measures (SAS^®^ PROC MIXED). Weeks (week 1 and week 4), times (T1 through T12), and treatment x time/treatment x week interaction were included as fixed effects while time and week were included as repeated effects. The total area under the curve (AUCs) of individual VAS appetite scores throughout the day (tAUCT1-T12) and for the times after consumption of the goûter (tAUCT5-T10) were calculated for each subject and goûter using the trapezoid method. A mixed model analysis of covariance (ANCOVA) for repeated measures (SAS^®^ PROC MIXED, SAS Institute Inc., Cary, NC, USA) was used to analyze the area under the curve (tAUC) for tAUC (T1–T12) and tAUC (T5–T10) for the appetite ratings. The Benjamini-Hochberg test was performed to adjust for multiple comparisons of significant effects to control the false discovery rate.

Statistical analyses were performed using SAS^®^ software version 9.3 (SAS Institute Inc., Cary, NC, USA). For all statistical tests (2-tailed), the 0.05 level of significance was used. Data are presented as means and standard deviations unless otherwise indicated.

### 2.6. Post hoc Analyses

A statistically significant higher daily energy intake was observed in the pistachio group as compared to the biscuit group in the run-in period (*p* < 0.0001). In order to take this baseline heterogeneity into account, a post hoc analysis of total daily energy intake (mean of the three-day food diary) in the run-in period was added as a second covariate in the ANCOVA for repeated measures.

## 3. Results

Sixty women completed the study. The mean age was 35 ± 7 years (range 23–49) and the mean BMI was 21.6 ± 1.72 kg/m^2^ (range: 18.8–25.0). The TFEQ of restrained eating was 5.6 ± 3.7 and the TFEQ score of disinhibition was 5.8 ± 2.78. Groups were homogeneous for age, BMI, anthropometrics, and body composition measurements (Table 2). However, the pistachio group had significantly higher (*p* < 0.0001) total daily energy intake than the biscuit group during the run-in period before dietary treatment started (Table 3). Most of this difference was due to a higher energy intake before the goûter (*p* < 0.0001). There was no significant difference in physical activity at either run-in or week four. The global compliance was 91.7%. For the biscuit group, compliance was 90.3% and for the pistachio group it was 93.1%. Nine women had low daily compliance (lower than 75% and one reported an adverse effect). Statistical results on the endpoints were similar independent of inclusion or exclusion of the values from these women. There was no statistical difference seen between groups on mean time of consumption of the afternoon goûter (21 ± 13 min and 27 ± 17 min for the biscuit and pistachio groups respectively).

There was no significant difference (*p* < 0.05) in evening energy intake (after the goûter) between the groups either at week one or at week four (Table 3). There was no significant difference within groups between run-in, week one, and week four in total energy intake. Although there was a decrease in total energy intake for the pistachio group, and an increase for the biscuit group between the run-in and week one, those differences did not reach significance (*p* = 0.061 for pistachio group change and *p* = 0.414 for the biscuit group change). There was no significant difference in total daily energy intake within groups at run-in, week one, and week four (*p* = 0.175 for the pistachio group change and *p* = 0.621 for the biscuit group change).

Anthropometric measurements remained stable throughout the study in spite of the goûters. There was no significant difference in body weight, waist and hip circumference, and waist/hip circumference ratio between run-in and week four in either group (Table 2). After four weeks, waist circumference tended to decrease among women who consumed pistachios. This decrease was not significant and it was not seen among women who consumed biscuits.

Neither consuming pistachios nor biscuits had an impact on body composition. There was no significant difference in fat and lean body mass and fat/lean body mass ratio between run-in and week four in either group (Table 2).

**Figure 2:**
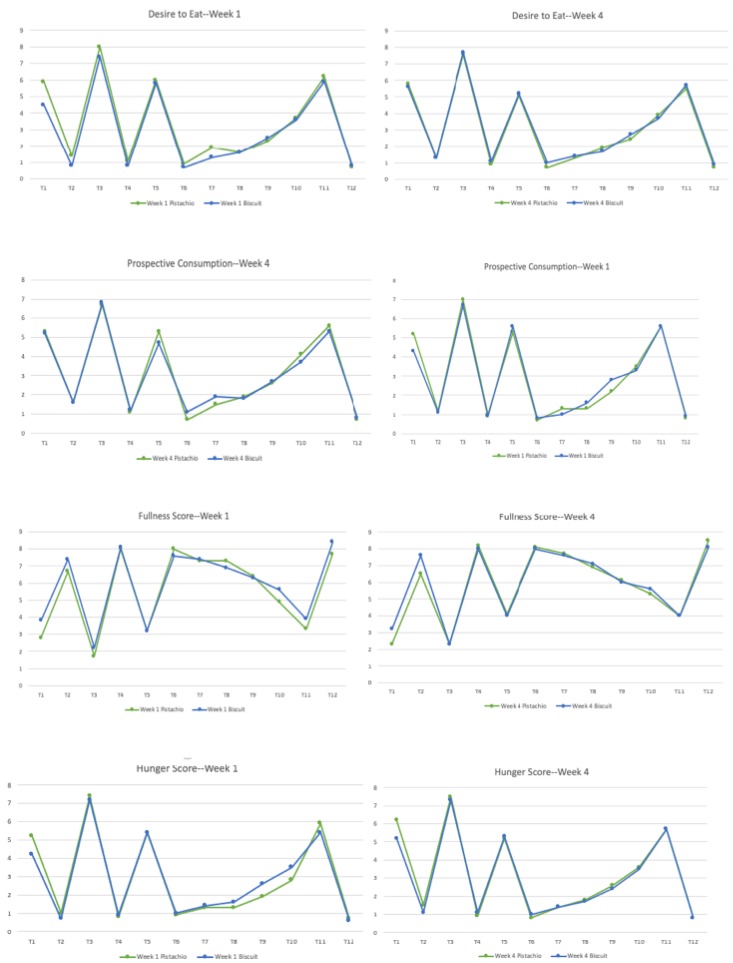
Mean visual analog scales (VAS) scores taken during the first week (week 1) and the last week (week 4) of the study. Time: Immediately before breakfast (T1) immediately after breakfast (T2), immediately before lunch (T3) immediately after lunch (T4), immediately before the goûter (T5) immediately after the goûter (T6), at 30 (T7), 60 (T8), 90 (T9), and 120 min after the goûter (T10), immediately before dinner (T11) and immediately after dinner (T12).

Subject ratings of satiety/appetite measures (desire to eat, hunger, fullness, and prospective consumption) obtained with the VAS were similar in the two groups (Figure 2). Some sporadic differences where noted. At week one, the decrease in hunger was greater 90 min after consuming pistachios than after consuming biscuits (*p* = 0.022). At week four, immediately after breakfast (T2), the score of fullness was significantly higher in the biscuit group as compared with the pistachio group (*p* = 0.048). At week one, immediately after breakfast (T2) the scores of hunger, desire to eat, and prospective consumption were higher in the pistachio group as compared with the biscuit group. There was no difference between the groups in VAS appetite rating scores expressed as tAUCT1-T12 or tAUCT5-T10 (Table 4).

Intakes of specific micronutrients were significantly higher at week one and week four for women who consumed pistachios compared with those who consumed biscuits (Table 3). Thiamin intake was on average 37% higher, vitamin B6 was 31% higher, copper intake was 68% higher, and potassium intake was 20% higher. There was no significant difference with respect to manganese and phosphorous intake.

## 4. Discussion

This is the first study to investigate the effect of an afternoon pistachio snack or goûter on next-meal self-reported energy intake and measures of satiety in French women, under free living conditions. There was no difference in evening energy intake or subjective ratings of satiety between women who consumed pistachios and those who consumed savory biscuits either at the beginning or at the end of the intervention study. Including a 1318 kJ goûter daily for four weeks did not affect body weight or composition. Women consuming the pistachio goûter, however, had significantly higher intake of vitamins and minerals that are particularly high in pistachios.

The goûter is a traditional small meal ingested in the afternoon by all French children and many adults. It usually involves no cooking or food preparation. Typical goûter options are ready-to-eat items, for example fruits, yogurts, pastries, chocolate. The goûter can be considered an afternoon snack because of its small size, relatively irregular nature, ready-to-eat options, and ingestion outside of main meals. Nevertheless, it can be considered a small meal when a person consumes it regularly. In our protocol, participants were regular consumers of goûter to fit with the time of day intended in the study.

Our results are consistent with previous reports where no difference in satiety measures was seen when comparing nuts to isoenergetic snacks. In a twelve-week study, Sayer et al. compared an afternoon snack of 28 g of almonds to a portion of baked food with equivalent energy and macronutrient content [42]. Postprandial hunger, desire to eat, and fullness assessed with VAS were not different either at the beginning or the end of the study. Rock et al. compared acute, postprandial satiety responses to isocaloric breakfast meals containing either walnuts or cream-cheese [50]. Appetite ratings (feelings of hunger, fullness, and anticipated prospective consumption) obtained with VAS were similar in response to the two meals. The acute effects of three high-fat meals on satiety were compared in a study by Casas-Agustench et al. using a crossover trial design and VAS measures. The meals were high in PUFA from approximately 47 g of walnuts, MUFA from olive oil or SFA from dairy. Satiety measures at 30, 90, 210, and 300 min postprandial was similar in all groups. In contrast, Brennan et al. found that isocaloric breakfast shakes containing 48 g of walnuts increased overall satiety and sense of fullness in pre-lunch questionnaires as compared with a placebo shake, matched in fat but not in protein content [40]. This was a four-day crossover study with a month washout period between treatments. The walnut effect achieved significance on day three and day four of testing. A similar amount of energy was consumed in all three test-days indicating that subjects compensated for the extra calories consumed. Tan et al., in a four-week randomized, parallel-arm study, found that a 43 g almond snack reduced hunger and desire to eat during the acute feeding session [37]. The control arm received no almonds. For all the studies mentioned above, test meals and satiety measures were administered during clinic visits.

The satiety value of nuts has been suggested as a potential mechanism to explain observations that nut consumption does not lead to weight gain and that including nuts in habitual diets is compatible with maintaining a healthy body weight [25]. The absence of a difference in post-goûter energy intake was not unexpected since the pistachio and the control goûter were closely matched for energy density and protein content. Energy density and protein content are important characteristics for mediating satiety response to foods. Kirkmeyer and Mattes showed that energy content may be the primary determinant on the impact of food on hunger [51]. Among the macronutrients, protein content is considered to be the most satiating [52]. Of consideration than, is the choice of an appropriate control – rather than choosing a popular snack food for the goûter, or no snack at all, we selected one that was closely matched in protein and energy content. This may have minimized differences in satiety effects between the groups.

Studies that compared a nut snack to no snack showed clear differences in satiety. For example, the acute effect of a mid-morning almond snack on satiety and subsequent food intake were assessed by Hull et al. [53]. For three days, 32 healthy women consumed a standard breakfast followed by 0, 28 or 42 g of almonds and then ad libitum meals at lunch and dinner. Satiety was assessed by measuring energy intake at the two ad libitum meals and subsequent appetite (VAS) throughout the test days. The energy intake of the participants decreased in a dose-response manner in response to the almond snack, and subjective appetite ratings in the interval between the snack and lunch were consistent with dose-dependent enhanced satiety.

In our study there was no difference in total daily energy intake between women who consumed pistachios and those who consumed biscuits at week one and week four. There was no significant difference in total daily energy intake within groups at run-in, week one, and week four. A meta-study on 23 randomized trials showed that adding nuts to the diet significantly increased daily energy intake [33]. Subgroup analyses based on the BMI of participants, however, showed that this was explained using the effect in overweight/obese individuals but not in individuals with normal weight. In these trials the amounts of nuts provided ranged from 20 g/day to 87.5 g/day. Daily energy intake was mostly assessed using three-day food records and control diets ranged from no nuts to commercial snacks, cereals or fruit.

In our study neither consuming pistachios nor biscuits had an impact on body weight or body composition after four weeks. Our study results are consistent with past studies where adding nuts to the diet did not cause weight gain [16,17,18,42,50,54,55], or influence weight loss during energy restriction [15,56,57]. Two recent meta-analyses on randomized clinical trials have examined the effect of nut consumption on body weight [33,58]. In one [33], assessment of weight was not among the primary aims but it examined studies which included weight among primary outcomes (15 studies). Nut consumption did not affect weight in the overall estimate. Li, on the other hand, in pooled data from 23 randomized trials, showed that a nut-supplemented diet was associated with a significant decrease in body weight and waist circumference [58]. Consistent with our study, a study on free-living, healthy women, adding 20% of the calories as pistachios for ten weeks did not affect body weight or body composition, but did increase diet quality [17]. The study was a randomized crossover study design conducted at two university campuses with two ten-week treatment periods and a fifteen-week washout period. In the pistachio treatment, energy intake was higher than the control (no pistachios).

Nuts, including pistachios, are nutrient dense foods, high in an array of vitamins and minerals. In observational studies, tree nut consumption is associated with better nutrient adequacy and higher diet quality [59,60,61]. In our study, women consuming the pistachio goûter had higher intakes of potassium, copper, manganese, thiamin, and vitamin B6, but not of phosphorous or manganese. It is worth noting that, other than potassium, intake of these micronutrients at run-in were within the recommended range in US and European dietary reference values for adult women [62,63]. Intervention studies consistently show that adding pistachios to the diet increases potassium levels [17,64]. Increasing potassium intake is a favorable change towards a heart healthy diet.

A limitation of the study could be the three-day food diary. This is a self-administered food intake record completed at home by the participants. In spite of precise instructions given to participants for its completion, some underreporting cannot be ruled out. Other limitations could be that the sample test consisted only of women who were normal in weight. It is possible that sex and weight status may influence the effect of nuts on the assessed variables [33]. While there was a high level of compliance, a small number of participants (nine) had low compliance. The statistical analyses, however, were similar even after exclusion of the nine women with low compliance. Finally, a four-week period is sufficient to assess maintenance of satiety effects over repeated presentations, but it may be too short to assess changes in body weight and composition. A strength of the current study includes a high retention rate (all participants completed the study).

More work is needed to establish the effect of pistachios on satiety. Data from this study may provide reliable information to be used in future studies with larger sample size and longer duration.

## 5. Conclusions

The consumption of pistachios or a savory biscuit of similar energy and protein content resulted in similar subsequent food consumption and subjective feelings of appetite and satiety. In our study a daily goûter of 1318 kJ (about 15% of energy intake) had no negative impact on body weight and composition. Women who consumed pistachios, however, had a higher intake of selected micronutrients. These findings confirm that pistachios, among other nuts, are a healthy snack option. Future work is needed with a larger sample size and longer-term studies.

## Figures and Tables

**Table 1:** Nutrient content of goûters.

	Roasted Lightly Salted Pistachios ^1^ (per 56 g serving)	Savory Cheese Biscuit (per 56 g serving) ^2^
Energy (kJ)	1318	1318
Water (g)	1.7	1.7
Proteins (g)	11.8	10.6
Carbohydrates (g)	10.6	24.6
Fibers (g)	5.5	1.2
Fat (g)	25	19
Saturated fats (g)	3.8	9
Mono-unsaturated fats (g)	14	ND
Poly-unsaturated fats (g)	7.3	ND
Phosphorous (mg)	263	140
Potassium (mg)	566	76
Sodium (g)	0.21	ND
Copper (mg)	0.72	0.06
Manganese (mg)	0.69	0.40
Thiamin (mg)	0.40	0.07
Vitamin B6 (mg)	0.63	0.03

^1^ U.S. Department of Agriculture, Agricultural Research Service. 2013. USDA National Nutrient Database for Standard Reference, Release 26. ^2^ Micronutrient analysis was conducted by Eurofins Food Testing UK Ltd., UK (12/04/2017). ND = not determined.

**Table 2:** Anthropometry and body composition at run-in and after four weeks of consuming either a pistachio or a savory biscuit goûter.

Variable	Run-in		Week 4		*p*
	Biscuit *N* = 30	Pistachios *N* = 30	Biscuit *N* = 30	Pistachios *N* = 30	Run-in-Week 4 Changes
Body weight (kg)	57.3 (5.8) ^1^	58.5 (6.0)	57.3 (5.5)	58.7 (6.6)	*p* = 0.9696
Waist circumference (cm)	74.8 (7.0)	74.2 (5.0)	74.7 (7.0)	73.6 (5.04)	*p* = 0.7202
Hip circumference (cm)	94.6 (5.2)	96.3 (5.6)	94.5 (5.1)	96.0 (5.3)	*p* = 0.1418
Waist/hip ratio	0.79 (0.0)	0.77 (0.0)	0.79 (0.0)	0.77 (0.0)	*p* = 0.9638
Fat body mas (%)	24.9 (6.0)	25.0 (3.6)	25.4 (6.1)	25.0 (3.6)	*p* = 0.2950
Lean body mass (%)	75.2 (6.0)	75.0 (3.6)	74.6 (6.1)	75.0 (3.6)	*p* = 0.2950
Fat/lean body mass	0.33 (0.1)	0.33 (0.1)	0.34 (0.1)	0.33 (0.1)	*p* = 0.2683

^1^ Mean (SD).

**Table 3:** Mean energy (kJ) and micronutrient intake (mg) throughout the study.

Variable (intake)	Run-in		Week 1		Week 4	
	Biscuit *N* = 30	Pistachios *N* = 30	Biscuit *N* = 30	Pistachios *N* = 30	Biscuit *N* = 30	Pistachios *N* = 30
Energy intake before goûter ^1^	3611 (706) ^5^ *	4542 (979) *	3880 (1094)	4234 (1491)	3942 (1096)	4290 (972)
Energy during afternoon goûter ^2^	787 (487)	1158 (1035)	1287 (289)	1242 (326)	1223 (383)	1338 (242)
Evening energy intake ^3^	2675 (846)	3005 (941)	2216 (871)	2403 (941)	2227 (731)	2610 (1383)
Total energy intake ^4^	7073 (1406) *	8705 (1586) ^*^	7383 (1510)	7860 (1843)	7410(1531)	8238 (1776)
Phosphorous	965 (222)	1076 (293)	1001 (235)	1083 (246)	975 (273)	1101 (241)
Potassium	2267 (756)	2503 (550)	2172 (461) †	2531(536) †	2165 (756) †	2654 (595) †
Copper	1.8 (1.94)	1.7 (0.67)	1.3 (0.40) †	2.0 (0.65) †	1.2 (0.40) †	2.2 (0.74) †
Manganese	3.2 (1.47)	4.0 (2.21)	3.3 (1.82)	3.8 (2.24)	3.3 (1.86)	3.9 (1.83)
Thiamin	1.0 (0.25)	1.2 (0.56)	0.9 (0.33) †	1.3 (0.37) †	1.0 (2.24) †	1.3 (0.46) †
Vitamin B6	1.3 (0.37)	1.6 (0.61)	1.3 (0.42) †	1.9 (56) †	1.3 (0.41) †	1.8 (0.56) †

* Significant difference between goûter types at run-in (*p* < 0.0001) and † significant difference between goûter types (*p* < 0.05) at week 1 and week 4. ^1^ Energy intake during breakfast + energy intake from morning snacks + energy intake during the afternoon (study food excluded); ^2^ energy by food, beverages during the afternoon goûter including study food; ^3^ energy intake during the dinner + post dinner intake; ^4^ eotal energy during the day including energy from goûter; ^5^ mean (SD).

**Table 4:** Mean subjective appetite responses from visual analogue scales after intake of biscuits or pistachio as goûter (T5–T10) and throughout the day (T1–T12) by women during the first and the last week of the study.

Variable (Intake)	Week 1		Week 4	
	Biscuit *N* = 30	Pistachios *N* = 30	Biscuit *N* = 30	Pistachios *N* = 30
AUC(T5-T10) ^1^ Hunger (a.u) ^3^	11.1 (5.7) ^4^	9.6 (5.9)	11.1 (5.5)	10.9 (5.9)
AUC(T5-T10) Fullness (a.u)	32.6 (9.0)	34.1 (6.5)	32.9 (8.2)	34.6 (9.1)
AUC(T5-T10) Desire to eat (a.u)	11.4 (5.0)	10.4 (6.4)	11.8 (6.9)	10.8 (6.8)
AUC(T5-T10) Prosp. Consumption (a.u)	11.0 (4.5)	9.9 (5.8)	12.0 (7.1)	11.3 (5.5)
AUC(T1-T12) ^2^ Hunger (a.u)	33.1 (9.0)	32.0 (9.1)	33.7 (12.0)	34.6 (11.5)
AUC(T1-T12) Fullness (a.u)	64.3 (12.8)	64.2 (9.4)	65.6 (13.4)	66.4 (11.4)
AUC(T1-T12) Desire to eat (a.u)	34.5 (8.3)	34.6 (9.1)	35.1 (12.6)	34.1 (12.9)
AUC(T1-T12) Prosp. Consumption (a.u)	32.8 (7.1)	32.3 (10.1)	34.1 (13.6)	33.6 (11.4)

^1^ tAUCT5-T10, area under the curve for the times after consumption of the goûter; ^2^ AUCT1-T12, total daily area under the curve of individual VAS scores; ^3^ arbitrary unit; ^4^ Mean (SD).
